# Reduction of the Heterocyclic Amines in Grilled Beef Patties through the Combination of Thermal Food Processing Techniques without Destroying the Grilling Quality Characteristics

**DOI:** 10.3390/foods10071490

**Published:** 2021-06-27

**Authors:** Wei Wang, Lu Dong, Yan Zhang, Huaning Yu, Shuo Wang

**Affiliations:** 1 Key Laboratory of Food Nutrition and Safety, Ministry of Education of China, Tianjin University of Science and Technology, Tianjin 300457, China; weilianyi@163.com; 2 Tianjin Key Laboratory of Food Science and Health, School of Medicine, Nankai University, Tianjin 300071, China; donglu@nankai.edu.cn (L.D.); yzhang@nankai.edu.cn (Y.Z.); 3 Midea Group Guangdong Midea Kitchen Appliances Manufacturing Co., Ltd., Foshan 528000, China

**Keywords:** heterocyclic amines, thermal food process, reduction, beef patties, quality

## Abstract

In order to reduce the formation of heterocyclic amines in grilled beef patties without destroying their unique quality characteristics, the effects of different thermal processes, including charcoal grilling, infrared grilling, superheated steam roasting and microwave heating, on the production of heterocyclic amines in beef patties and grilling quality characteristics were systematically analyzed. The results showed that infrared grilling can significantly (*p* < 0.05) reduce the content of heterocyclic amines in grilled patties, and the combination of microwave heating or superheated steam roasting with infrared grilling could further reduce the content of heterocyclic amines, with a maximum reduction ratio of 44.48%. While subtle differences may exist in infrared grilled patties with/without superheated steam roasting or microwave heating, a slight change will not affect the overall quality characteristics of grilled patties. The combined thermal processing will not visually affect the color of the grilled patties. Correlation analysis and regression analysis showed that the reduction in heterocyclic amines caused by microwave heating and superheated steam roasting are related to the moisture content and lipid oxidation of grilled patties, respectively. Using combined thermal processes to reduce the formation of heterocyclic amines is advisable.

## 1. Introduction

Carcinogenic heterocyclic amines (HAs) are formed in protein-rich foodstuffs (e.g., meat) during thermal processes such as grilling. HAs are classified into two major types: thermic HAs (amino-imidazo-azaarenes (AIAs)) and pyrolytic HAs (amino carbolines). Thermic HAs (such as 8-MeIQx (2-amino-3,8-dimethylimidazo [4,5-ƒ]quinoxaline), 4,8-DiMeIQX (2-amino-3,4,8-trimethylimidazo[4,5-f]quinoxaline), and PhIP (2-amino-1-methyl-6-phenyl-imidazo[4,5-b]pyridine)) are commonly formed by amino acids, creatinine (creatine) and glucose due to the Maillard reaction, while pyrolytic HAs (such as Norharman (9 H-pyrido[4,3-b]indole) and Harman (1-methyl-9 H-pyrido[4,3-b]indole)) are produced by the pyrolysis of proteins [1]. The formation of HAs in food was influenced by the type, temperature and duration of the thermal processes [2].

The formation of HAs by adjusting thermal processes or process conditions and adding with antioxidant compounds can be mitigated [3]. Adding active substances to foodstuffs has attracted extensive interest because of its convenience. However, it is undeniable that the thermal instability of the active substance limits its effect [4], and may introduce new, potentially harmful compounds which were formed by complex reactions between the new compounds and the composition of meat [5,6].

Charcoal grilling (CG) is a traditional cooking method that can produce HAs, and the scorching phenomenon of CG affects people’s appetite due to uneven heating [7]. In terms of the quality of the products, infrared grilling (IG) is an effective technology due to its uniform and faster heating [8]. Compared with IG, microwave heating is more efficient because it can penetrate to deeper parts of the food, while almost all the energy of infrared radiation is absorbed by the surface of the food, and slow heat conduction needs to enter the food for infrared radiation [9]. Although the microwave has been widely used in food processing, it has several disadvantages. One is that many foods cannot trigger a browning reaction through microwaves, because the temperature of the food surface is lower than the inside, but it can be used in combination with IG to heat the food surface to a high enough temperature to cause a browning reaction. The second is that the cooked meat loses more moisture and the texture of the meat is too dry due to the rapid heating, which can be improved by combining this with superheated steam roasting (SHS). SHS is a modern thermal process that can increase the heat penetration rate in meat products when the saturated steam continues to be heated at a given pressure, since the temperature of the steam is higher than the saturation temperature [10].

When studying the thermal food processing techniques, researchers like to compare techniques in isolation to study the production of HAs [11,12,13]. The bottleneck of the research on the influence of processing methods on HAs is that few people have combined several processing methods into the same thermal processing to reduce and control the production of HAs. Furthermore, research on the influence of the microwave on the content of HAs has focused on the microwave as a pretreatment method [14,15], and the effect of microwave cooking on the formation of HAs is still unclear. In a study by Oz et al. [13], microwave cooking could reduce the formation of HAs in fried chicken meat products, whereas another study found higher mutagenic activity in meat cooked by the microwave [9,16]. Whether the microwave can reduce HAs remains to be determined. Moreover, there is not much research on the influence of SHS on the content of HAs [17,18], and explorations of the possible causes connected with other substrates in food are rare. Our goal was to reduce the production of carcinogenic HAs by a combination of thermal processing techniques, and to offer preliminary explorations for this. In this process, we also ensured the quality characteristics of the patties.

To realize the reduction in the formation of HAs, and maintain the original grilling characteristics of grilled patties, the effects of different thermal processes with different heating principles and characteristics and their combinations on HAs formation, as well as the quality characteristics (texture, color), moisture content and lipid oxidation (thiobarbituric acid (TBA) value) of beef patties were investigated. Additionally, the correlation and regression analyses between HAs, moisture content and the TBA value of grilled patties were analyzed to explore the involved mechanisms, which will provide a new strategy for the mitigation of unhealthy compounds and improve the safety and quality of thermal processed foods.

## 2. Materials and Methods

### 2.1. Materials and Reagents

Lean shoulder blade beef meat was obtained from the local supermarket (Tianjin, China). All analytical grade chemicals including ammonia, hydrochloric acid, sodium hydroxide, magnesium sulfate, trichloroacetic acid, chloroform, ethylene diamine tetraacetic acid (EDTA) and acetate were obtained from Toronto Research Chemicals Co. CAN. The HPLC-grade solvents including methanol and acetonitrile, and 11 HAs standards (˃99% purity) including MeIQ (2-Amino-3,4-dimethylimidazo[4,5-ƒ]quinoline), IQx (2-amino-3-methylimidazo[4,5-f]-quinoxaline), 8-MeIQx, 4,8-DiMeIQx, 7,8-DiMeIQx (2-amino-3,7,8-trimethylimidazo[4,5-f]quinoxaline), PhIP, Harman, Norharman, Trp-P-2 (3-Amino-1-methyl-5 H-pyrido[4,3-b]indole), AαC (2-Amino-9 H-pyrido[2,3-b]indol) and MeAαC (2-Amino-3-methyl-9 H-pyrido[2,3-b]indol) were purchased from Sigma Aldrich Co. USA. Oasis^®^ MCX 3 cc (extraction column) and ACQUITY UPLC^®^ BEH C18 (2.1 mm × 100 mm, 1.7 μm) were procured from Waters Corporation Co. USA.

### 2.2. Sample Preparation

The lean meat was thawed over-night (5 °C) and then chopped using a mechanical mincer. No salt and other spices were applied to the minced meat. Every 100 ± 1 g of meat was made into each patties sample through the mold (100 mm dia. × 15 mm thick). Next, 66 beef patties samples were prepared for each batch and three batches of beef patties samples were grilled in total.

### 2.3. Cooking of Patties by Different Thermal Processes

Beef patties were grilled according to five grilling processes (CG (charcoal grilling), IG (infrared grilling), IG-SHS (superheated steam roasting)-IG, IG-microwave-IG, IG-microwave-SHS-IG). Three replicates were performed for each thermal process. For CG, the patties were grilled over charcoal which was placed in the bottom of the charbroiler (CF-E112013, e-Rover Ltd., Ningbo, China). In addition, the grilled beef patties preparation oven is an internal modification equipment in the laboratory, which integrates the three functions of IG, SHS and microwave heating. The equipment parameters are: the size of the oven cavity is 380 mm × 320 mm × 200 mm; the grilling temperature range is 0–230 °C; the microwave output power range is 200–1000 W; the superheated steam roasting temperature is 100 °C. For combined thermal processes, the patties were grilled for 8 min with IG first to ensure the grilled flavor of the patties. The 8-min grilling temperature in the first step was consistent with the grilling temperature used in the subsequent grilling process. In Table 1, Table 2 and Table 3, the grilling temperature of each combined thermal process was marked. The grilling tray inside the oven is grid-shaped so that patties can be grilled on both sides at the same time. Three grilling temperatures (180 °C, 200 °C and 220 °C), two values of microwave power (1000 W, 500 W), three values of microwave time (10 s, 30 s and 60 s) and three SHS times (3, 4 and 5 min) were studied. All the thermal processes were ended when patties reached an internal temperature of 75 °C, which was monitored using a thermocouple thermometer (4 CH-20 CH, GND Ltd., Shanghai, China).

### 2.4. Quantification of HAs

To obtain good recovery and reproducibility, HAs were purified on Oasis MCX cartridge (Polypropylene, 3 cc, 60 mg), then analyzed by UPLC-MS/MS (ACQUITY/Xevo TQ-S micro, Waters, Milford, MA, USA) method. In brief, 1 g of cooked meat sample was homogenized in a 10 mL NaOH solution (0.1 M) for 5 min. Then, 10 mL of acetonitrile and vortex was added for 1 min, followed by the addition of 6 g MgSO_4_ and 1.5 g sodium acetate and vortex for 5 min. Finally, this was centrifuged at 5000 r/min for 5 min, and the supernatant was collected for solid phase extraction. The MCX cartridge was activated with 3 mL methanol and 3 mL 0.01 M hydrochloric acid solution, and the collected supernatant was loaded on the activated MCX column; then, 3 mL 2% formic acid water, 9 mL deionized water and 3 mL methanol were used to wash the impurities. A total of 3 mL of mixture of methanol and ammonia (9:1, *v*/*v*) was used as the eluent, then the eluent containing the HAs was collected and dried using a nitrogen gas stream, before being reconstituted with 1 mL of methanol. It was filtered through a 0.22 μm organic microporous filter membrane into a sample vial for UPLC-MS/MS analysis.

An ACQUITY UPLC BEH C18 column was used for the separation. The column temperature was set to 45 °C. The mobile phase, A and B, was made up of ultrapure water and acetonitrile. The gradient programm was: 0–0.5 min, 90% A; 0.5–10 min, 50–90% A; 10–10.1 min, 50–90% A; 10–15 min, 90% A. An optimal baseline separation was achieved at a flow rate of 0.5 mL/min. ESI was performed in positive-ion mode; MRM mode was used for MS/MS detection. The optimal ionization source working parameters were as follows: nebulizer pressure, 40 psi; and capillary voltage, 900 V. The drying gas flow rate and temperature were 800 L/Hr and 400 °C, the cone gas flow rate was 60 L/Hr, and the ion source temperature was 150 °C. The limits of detection (LODs), as well as the limits of quantitation (LOQs) and recovery of UPLC-MS/MS, are given in Supplementary Table S1.

### 2.5. Moisture Content Analysis

The moisture content of grilled patties was measured according to the China National Standard method (GB5009.3, 2016).

### 2.6. Determination of TBA Value

The TBA values were determined by the Method of Hai et al. [19]. A total of 50 mL of 7.5% trichloroacetic acid (containing 0.1% EDTA) was added to 10 g of the homogenized meat sample and shaken for 30 min. Thereafter, the preparation was filtered twice with filter paper, and 5 mL of the supernatant was treated with 5 mL of 0.02 mol/L TBA solution, incubated in a 95 °C water bath for 50 min, cooled to room temperature and centrifuged for 5 min (10,000 r/min). The supernatant obtained was treated with 5 mL of chloroform and centrifuged for 5 min (10,000 r/min). Finally, the supernatant underwent colorimetric analysis at 532 and 600 nm (Evolution 350, Thermo Fisher, Waltham, MA, USA). The absorbance was recorded and a blank test was performed. The results are expressed as milligrams of MDA per kilogram of meat (mg MDA/Kg).

### 2.7. Color Analysis

Color parameters were measured and the values were recorded as three parameters of lightness (L*), redness (a*), and yellowness (b*). The surface of the patties was randomly tested on by a chroma meter (CR-210, Minolta Ltd., Tokyo, Japan).

### 2.8. Texture Profile Analysis

The beef patties were cut into cubes (1 cm × 2 cm × 2 cm) and tested with a Texture Analyzer (TA-XT2 i, SMS Ltd., London, UK). Three cubes (1 cm × 2 cm × 2 cm) were taken from each sample, and nine cubes were totally taken from three parallel samples of each thermal treatment for determination. Samples were compressed to 30% of their original height by a cylindrical probe with a 3 cm diameter and 5 mm/s speed. The parameters of hardness, springiness, cohesiveness, gumminess, chewiness and resilience were recorded.

### 2.9. Statistical Analysis

The data are expressed as the means ± standard errors. SPSS (v. 24.0) was used for difference analysis by Duncan test and correlation analysis by Two-tail test. Origin Pro (v. 2019) was used for drawing figures.

## 3. Results and Discussion

### 3.1. Effects of Different Thermal Processes on the Production of HAs in Grilled Beef Patties

The effect of different thermal processes on the production of HAs in grilled beef patties was shown in Table 4. The overall amount of HAs in charcoal grilled beef patties was 1.093 μg/kg, which was higher in HAs content than patties grilled in the oven due to the production of thermic HAs (AIAs, about 55%). For pyrolytic HAs, only Harman and Norharman were detected, as Felton et al. [20] believe that although Harman and Norharman can be formed, other pyrolytic HAs are basically undetectable in processed meat products. For thermic HAs, the content of PhIP, 8-MeIQX and 4,8-DiMeIQX were under the limit of detection in patties grilled in the oven. This finding is the same as the study by Polak et al. [21], who believe that most HAs are formed rapidly after heating, and degrade after reaching the highest level. Since the surface of patties is in contact with the highest temperature, HAs are easily produced on the surface of patties. As a type of polar HAs, AIAs can diffuse into the inside of patties, where the temperature is much lower, which can prevent self-decomposition by heat. The patties grilled by CG were exposed to charcoal fire and resulted in a higher conductive heat transfer and the shortest thermal processing time (The cooking times of the thermal processes are given in Supplementary Table S2). The relatively short thermal processing time of CG was not sufficient for AIAs to undergo thermal decomposition, so that part of the AIAs remained in the patties.

HAs produced by IG-SHS-IG patties were fewer than those produced by IG patties, which is consistent with the study by Suleman et al. [22]. The three superheated steam times added when the grilling temperature is 200 °C can significantly reduce the total amount of HAs; the inhibition rate of total HAs reaches 44.48% when the superheated steam time is 4 min, which is the most effective process. Pathare and Roskilly [23] consider that SHS can reduce the surface temperature of the product and distribute heat evenly, thereby reducing the amount of HAs.

Similarly, HAs produced by IG-microwave-IG patties were lower (*p* < 0.05) than that produced by IG (200 °C) patties. Among the four time and power combinations, the total HAs reduction was as high as 11.49% (500 W 10 s) to 44.48% (1000 W 10 s). The amount of HAs decreased with the extension of microwave time and a decrease in microwave power. Furthermore, the successive addition of microwave and SHS will also reduce the amount of HAs. However, in terms of the inhibition of HAs, the continuous use of the microwave and SHS in one process was not as effective as adding microwave or SHS alone to IG. Compared with the 0.377 ± 0.046 μg/kg HAs produced by IG-SHS (4 min)-IG (200 °C) patties, HAs produced by IG-microwave-SHS (4 min)-IG (200 °C) patties was increased. The combination of microwave and SHS was not synergistic in inhibiting the production of HAs perhaps due to different inhibition reasons.

### 3.2. Effects of Different Thermal Processes on the Moisture Content in Grilled Beef Patties

**Figure 1:**
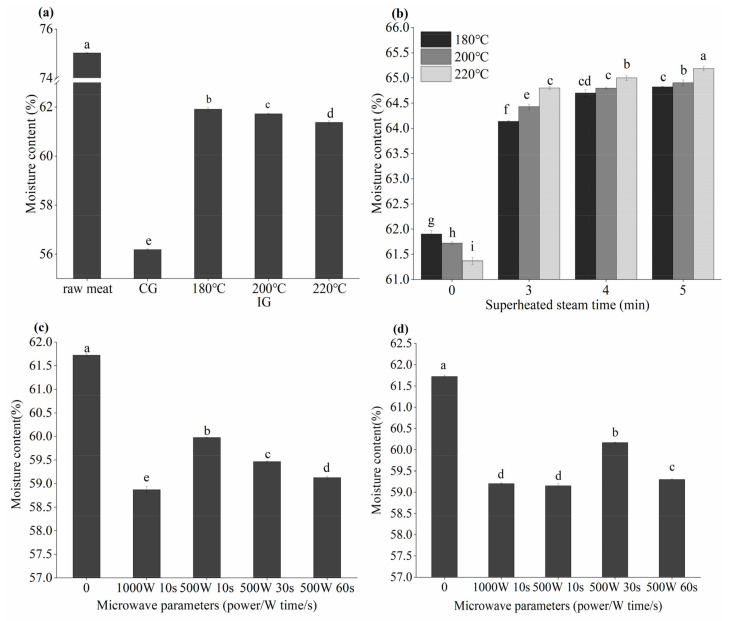
Moisture content in beef patties with different thermal processes. “CG” means charcoal grilling, “IG” means infrared grilling, and “SHS” means superheated steam roasting. (**a**) CG and IG (180, 200, 220 °C); (**b**) IG and IG-SHS-IG, the abscissa “0” represents IG; (**c**) IG (200 °C) and IG-microwave-IG (200 °C), the abscissa “0” represents IG (200 °C); (**d**) IG -SHS (4 min)-IG (200 °C) and IG-microwave-SHS (4 min)-IG (200 °C), the abscissa “0” represents IG-SHS (4 min)-IG (200 °C). Different small letters indicate significant difference *p* < 0.05 between different cooking methods.

The effect of different thermal processes on the moisture content in grilled beef patties is shown in Figure 1.

The moisture content of raw meat was 75.03%. Among the 21 cooking methods, the lowest moisture loss was 13.11% (IG (8 min)-SHS (5 min)-IG (220 °C)) and the highest was 25.12% (IG (8 min)-microwave (1000 w 10 s)-IG (200 °C)).

IG-SHS-IG patties had higher moisture content than samples grilled by other methods. As a new type of drying technology, SHS can take away the moisture in the food; however, due to the small drying stress, it can retain some moisture [24]. Drying stress is an important parameter to maintain product quality during the drying process. Too much drying stress will cause the product to lose too much water and cause dry cracking [25].

Compared with IG (220 °C) patties, the moisture content of IG-microwave-IG (200 °C) patties were significantly (*p* < 0.05) reduced, and the moisture content decreased with the extension of the microwave time from 59.98% to 59.13%. Additionally, the moisture content of IG-microwave-SHS (4 min)-IG (200 °C) patties was decreased compared to IG-SHS (4 min)-IG (200 °C) patties, indicating that the influence of microwave on the moisture of patties is greater than that of SHS.

### 3.3. Effects of Different Thermal Processes on the TBA Value in Grilled Beef Patties

**Figure 2:**
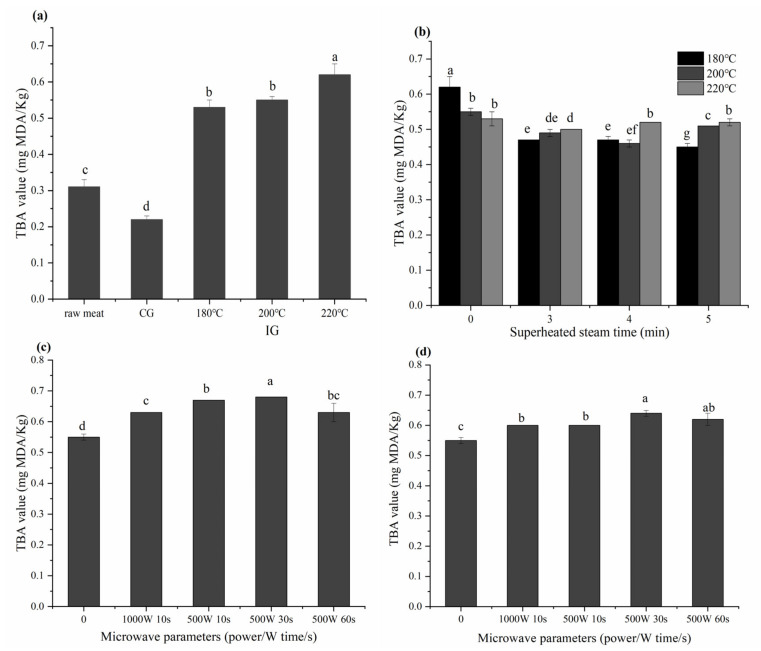
TBA values in beef patties with different thermal processes. “CG” means charcoal grilling, “IG” means infrared grilling and “SHS” means superheated steam roasting. (**a**) CG and IG (180, 200, 220 °C); (**b**) IG and IG-SHS-IG, the abscissa “0” represents IG; (**c**) IG (200 °C) and IG-microwave-IG (200 °C), the abscissa “0” represents IG (200 °C); (**d**) IG-SHS (4 min)-IG (200 °C) and IG-microwave-SHS (4 min)-IG (200 °C), the abscissa” 0” represents IG-SHS (4 min)-IG (200 °C). Different small letters indicate the significant difference *p* < 0.05 between different cooking methods.

The effect of different thermal processes on the TBA value in grilled beef patties is shown in Figure 2.

Lipid oxidation in meat products is usually evaluated according to the TBA value, using the method for the determination of 2-thiobarbituric acid reactive substances [26]. The TBA value of cooked patties was risen, except for CG patties. For CG, the higher temperature and the direct contact with patties may cause the lipid oxidation products to react with other molecules such as amino acids, reducing the amount of oxidation products such as malondialdehyde (MDA) [27]. For IG, the TBA value increased by about 1 time. As the grilling temperature increased, the content of MDA was risen.

The TBA value of IG-SHS-IG patties was decreased. The TBA value of IG-SHS (5 min)-IG (180 °C) patties was 0.45 mg MDA/Kg; at this time, the lipid oxidation degree of patties was the lowest. Studies have shown that SHS causes food to produce fewer free radicals and can reduce lipid oxidation in food [28]. A study by Wang et al. [29] showed that superheated steam makes the content of oxidation products (e.g., aldehydes) significantly lower compared to hot air drying.

The TBA value of IG-microwave-IG patties was decreased. For IG-microwave (500 w 30 s)-IG (200 °C), the TBA value of patties is 0.68 mg MDA/Kg, which is about 1.3 times higher than that of raw meat. Rababah et al. [30] found that the TBA value of microwave-cooked lamb was higher than that of lamb cooked in a traditional electric oven. Similarly, Weber et al. [31] believe that the degree of oxidation in microwave samples is higher than that in oven-grilled samples. Certain interactions between microwave and meat lipid lead to the accelerated oxidation of polyunsaturated fatty acids, increasing the secondary oxidation products derived from these fatty acids [32,33].

### 3.4. Correlation Analysis and Regression Analysis of HAs with TBA Value and Moisture Content of Grilled Beef Patties

**Figure 3:**
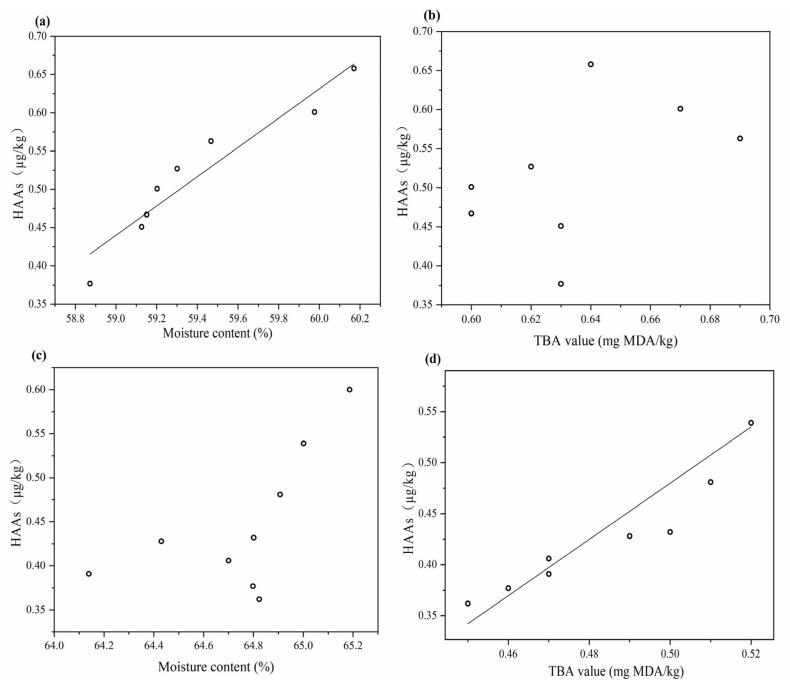
Linear regression lines between total HAs, TBA value and moisture content in patties grilled combined with microwave or SHS (superheated steam roasting). Microwave: (**a**,**b**), SHS: (**c**,**d**).

The Pearson coefficients between hazards and TBA values, as well as the moisture content in patties grilled combined with microwaves or SHS, are shown in Table 5. Linear regression lines between total HAs, TBA values and moisture content in grilled patties, combined with microwaves or SHS, are shown in Figure 3.

The Norharman produced in patties grilled combined with microwave was significantly positively correlated to the moisture content (0.936, *p* < 0.01). Regression analysis showed that the total amount of HAs of cooked patties subjected to microwaving increased linearly with the moisture content of the patties. For microwaving, moisture plays a major role in the formation of HAs. Persson et al. [34] believes that water is the reaction and transmission medium in the heating process for meat products. Water can transport precursor substances to the surface of the patties. On the surface, these substances are exposed to higher levels, and conducive to the formation of HAs at high temperatures. Persson et al. [35] added a mixture of tripolyphosphate and sodium chloride to minced meat to improve water retention, and Shin et al. [36] added carbohydrates to minced meat to bound water, resulting in reduced HAs formation. The key to heating food with electromagnetic waves (e.g., microwave and infrared radiation) lies in matching the wavelength and the energy level of the molecules in the food. Due to the different frequencies of infrared radiation and microwave, the frequency at which the object molecules resonate is also different. The frequency of household microwaves is 2450 MHz, closer to the vibration frequency of water molecules (2500 MHz) in food. Therefore, the natural frequency of microwave is more suitable for the high-frequency vibration of water molecules in food, which make it difficult for water molecules to transfer from the inside to the surface of the patties, so precursors of HAs will not easily transfer to the surface of the patties. Furthermore, the consumption of water molecules results in a higher droplet volume in the patties; that is, the precursors drip into the oven with the moisture easily during the heating process, resulting in lower HAs, as confirmed by Soladoye et al. [37]. In addition, the microwave wavelength is shorter than infrared and it is not easy to diffract. Therefore, the penetration ability is stronger than infrared radiation. Infrared can only heat the food surface, while the microwave can penetrate several centimeters to generate heat inside the food and avoid local overheating on the food surface, which inhibits the production of HAs.

For SHS, lipid oxidation plays a major role in the formation of HAs. The TBA value was significantly positively correlated to the Norharman (0.934, *p* < 0.01) and Harman (0.754, *p* < 0.05) produced in the patties grilled combined with SHS. Regression analysis showed that the total HA amount for patties grilled combined with SHS increased linearly with the TBA value of the patties. Studies have shown that the free radicals and intermediates produced by lipid oxidation significantly promote the formation of HAs [38,39,40,41]. Johansson et al. [42] used antioxidants such as tocopherol to inhibit lipid oxidation and the formation of HAs. Zamora et al. [43] found that the formation level of HAs doubled by adding iron ions to the model system due to the iron-catalyzed lipid peroxidation and the formation of free radicals. In our study, SHS can reduce the formation of HAs by reducing the degree of lipid oxidation in patties. Similarly, Kondjoyan et al. [44] believed that the absence of oxygen during SHS would minimize the degree of lipid oxidation, while reducing the level of HAs detected in beef slices. The intermediate product produced by the reaction of tryptophan with acetaldehyde and formaldehyde undergoes a Pictet–Spengle reaction to form a closed ring, and then the intermediates undergo multiple oxidations to produce Norharman and Harman [45]. The free radicals and oxidation products produced by lipid oxidation may participate in the oxidation process and promote the formation of Norharman and Harman. This mechanism needs to be further explored.

### 3.5. Effects of Different Thermal Processes on Quality Characteristics in Grilled Beef Patties

#### 3.5.1. Effects of Different Thermal Processes on the Color of Grilled Beef Patties

**Figure 4:**
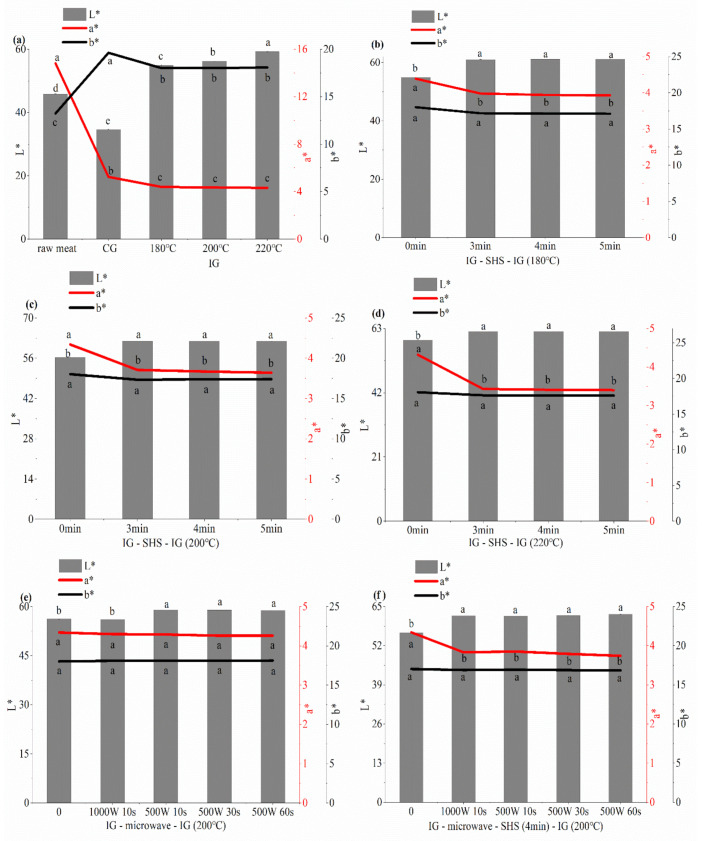
The effect of different thermal processes on the color in grilled beef patties. “CG” means charcoal grilling, “IG” means infrared grilling, and “SHS” means superheated steam roasting. (**a**) CG and IG (180, 200, 220 °C); (**b**) IG (180 °C) and IG-SHS-IG (180 °C), the abscissa “0” represents IG (180 °C); (**c**) IG (200 °C) and IG-SHS-IG (200 °C), the abscissa “0” represents IG (200 °C); (**d**) IG (220 °C) and IG-SHS-IG (220 °C), the abscissa “0” represents IG (220 °C); (**e**) IG (200 °C) and IG-microwave-IG (200 °C), the abscissa “0” represents IG (200 °C) (**f**) IG -SHS (4 min)-IG (200 °C) and IG-microwave-SHS (4 min)-IG (200 °C), the abscissa “0” represents IG-SHS (4 min)-IG (200 °C). Different small letters indicate significant difference *p* < 0.05 between different cooking methods.

The effect of different thermal processes on the color in grilled beef patties are shown in Figure 4. L*, a* and b* represent brightness, redness and yellowness values, respectively. A higher degree of myofibril changes and sarcoplasmic protein aggregation can increase the degree of light scattering and the L* value of patties. Maillard reaction can produce melanoid pigment substances, resulting in a decrease in the L* value and an increase in the b* value [29]. In our study, the color of charcoal grilled patties was difficult to control and reached the late heating stage quickly, creating the lower L* value. The a* value of patties is related to the conversion between myoglobin, oxymyoglobin and metmyoglobin. The a* value of charcoal grilled patties was higher than that of patties grilled in the oven, because the patties were exposed to sufficient oxygen to accelerate the production of bright red oxymyoglobin in the patties. Because the higher ambient temperature accelerated the Maillard reaction and increased the browning of the patties, the b* value of the charcoal grilled patties was higher than that of the patties cooked in the oven.

Due to the higher humidity in the oven and the increase of the moisture content on the surface of IG-SHS-IG patties, the rate of oxidation of myoglobin to oxygenated myoglobin was slowed down and the degree of light scattering on the surface of the patties was increased. IG-SHS-IG patties had a higher L* value and lower a* values than by IG patties, but the variation in amplitudes was small. Compared with IG (200 °C) patties, the addition of 500 W power microwave slightly increased the L* value of patties, but there is no significant difference between the a* and b* values (*p* > 0.05). The addition of a 1000 W power microwave did not change the color of infrared grilled patties. In addition, the microwave times had no effect on the color difference of infrared grilled patties. Adding a microwave to IG will better maintain the color of the infrared grilled patties. The combined thermal processes selected in this study did not visibly influence the color of grilled patties.

#### 3.5.2. Effects of Different Thermal Processes on the Texture of Grilled Beef Patties

The effect of different thermal processes on the texture in grilled beef patties was shown in Table 6. Different heat transfer methods lead to different heat penetration rates, which affects the formation rate of myofibril protein gel [46]. The hardness of charcoal grilled patties was much greater than with other cooking methods. The hardness, springiness and chewiness of the infrared grilled patties increased with an increasing cooking temperature (*p* < 0.05). According to our research, CG is not conducive to controlling the texture of the patties, while IG can control the texture of the patties by controlling the grilling temperature, which makes the operation more flexible. For the combined thermal processes, the SHS conditions can better maintain the grilled texture of the patties than microwave heating. Although the hardness, springiness and chewiness of IG-SHS-IG patties were increased at all the cooking intervals, this change was slight, from 4969.90 (the hardness value of IG (200 °C)) to 5061.81 (the hardness value of IG-SHS (4 min)-IG (200 °C)). For microwave, the hardness value was from 4969.90 (IG (200 °C)) to 5289.27 (IG-microwave (500 w 10 s)-IG (200 °C)). A change of less than an order of magnitude will not affect the grilled texture of the patties, nor will it affect consumer acceptance.

## 4. Conclusions

Of all the thermal processes involved in this research, the greatest moisture loss, highest values of dark color and highest HA content were observed in charcoal grilled beef patties. Compared with CG, AIA-type heterocyclic amines obtained by other thermal processes in the oven are easily degraded due to the prolonged grilling time. IG combined with microwave or SHS, as well as the successive use of three technologies, effectively decreased the content of HAs. Additionally, the combination of thermal processes can ensure the grilled quality of beef patties, and the combined use of SHS technology has a lesser impact on the texture of the patties compared to the combined use of microwave technology. Regarding color, the yellowness value of the patties grilled by the combined thermal processes did not change compared with the patties grilled by IG, but the brightness value of the patties is easily affected by the combined grilling processes. The combined use of microwave technology and IG has a lesser impact on the color of the patties compared with the combined use of SHS and IG. It is difficult for people to visually distinguish this slight color change. In addition, compared with IG, the microwave consumes more moisture and accelerates the lipid oxidation of patties, but SHS can better retain the moisture of patties better and reduce the degree of lipid oxidation in patties. Correlation analysis and regression analysis show that the inhibitory effects of a microwave and SHS on the formation of HAs are related to the moisture content and lipid oxidation of grilled patties, respectively. The above results showed that IG combined with SHS or a microwave could be a superb alternative to maintain the quality profiles and control the HAs of grilled patties, but this mechanism needs to be further explored.

## Figures and Tables

**Table 1:** Thermal process of “IG-SHS-IG”. “IG” means infrared grilling, “SHS” means superheated steam roasting.

Cooking Process	IG		SHS		IG
IG (8 min)-SHS (3 min)-IG (180 °C)	180 °C	8 min	180 °C	3 min	grill at 180 °C until the center temperature of the patties is 75 °C
IG (8 min)-SHS (4 min)-IG (180 °C)				4 min	
IG (8 min)-SHS (5 min)-IG (180 °C)				5 min	
IG (8 min)-SHS (3 min)-IG (200 °C)	200 °C		200 °C	3 min	grill at 200 °C until the center temperature of the patties is 75 °C
IG (8 min)-SHS (4 min)-IG (200 °C)				4 min	
IG (8 min)-SHS (5 min)-IG (200 °C)				5 min	
IG (8 min)-SHS (3 min)-IG (220 °C)	220 °C		220 °C	3 min	grill at 220 °C until the center temperature of the patties is 75 °C
IG (8 min)-SHS (4 min)-IG (220 °C)				4 min	
IG (8 min)-SHS (5 min)-IG (220 °C)				5 min	

**Table 2:** Thermal process of “IG–microwave-IG”. “IG” means infrared grilling.

Cooking Process	IG	Microwave Heating		IG
IG (8 min)-microwave (1000 w 10 s)-IG (200 °C)	200 °C, 8 min	1000 W	10 s	grill at 200 °C until the center temperature of the patties is 75 °C
IG (8 min)-microwave (500 w 10 s)-IG (200 °C)		500 W	10 s	
IG (8 min)-microwave (500 w 30 s)-IG (200 °C)			30 s	
IG (8 min)-microwave (500 w 60 s)-IG (200 °C)			60 s	

**Table 3:** Thermal process of “IG-microwave-SHS-IG”. “IG” means infrared grilling, “SHS” means superheated steam roasting.

Cooking Process	IG	Microwave Heating		SHS	IG
IG (8 min)-microwave (1000 w 10 s)-SHS (4 min)-IG (200 °C)	200 °C, 8 min	1000 W	10 s	200 °C, 4 min	grill at 200 °C until the center temperature of the patties is 75 °C
IG (8 min)-microwave (500 w 10 s)-SHS (4 min)-IG (200 °C)		500 W	10 s		
IG (8 min)-microwave (500 w 30 s)-SHS (4 min)-IG (200 °C)			30 s		
IG (8 min)-microwave (500 w 60 s)-SHS (4 min)-IG (200 °C)			60 s		

**Table 4:** Effects of different thermal processes on the production of HAs in grilled beef patties.

Cooking Process	Types of HAs (μg/kg)					Total HAs
	8-MeIQX	Norharman	4,8-DiMeIQX	Harman	PhIP	(μg/kg)
CG	0.320 ± 0.061	0.242 ± 0.040	0.118 ± 0.020	0.247 ± 0.014	0.166 ± 0.010	1.093 ± 0.090
IG (180 °C)	ND	0.355 ± 0.052 ^cd^	ND	0.088 ± 0.011 ^bc^	ND	0.443 ± 0.061 ^cd^
IG (200 °C)	ND	0.544 ± 0.013 ^aAx^	ND	0.136 ± 0.036 ^bcAx^	ND	0.679 ± 0.048 ^abAx^
IG (220 °C)	ND	0.501 ± 0.15 ^ab^	ND	0.235 ± 0.12 ^a^	ND	0.736 ± 0.27 ^a^
IG (8 min)-SHS (3 min)-IG (180 °C)	ND	0.297 ± 0.033 ^d^	ND	0.094 ± 0.016 ^bcA^	ND	0.261 ± 0.23 ^d^
IG (8 min)-SHS (3 min)-IG (200 °C)	ND	0.332 ± 0.028 ^cd^	ND	0.096 ± 0.021 ^bc^	ND	0.428 ± 0.042 ^cd^
IG (8 min)-SHS (3 min)-IG (220 °C)	ND	0.335 ± 0.072 ^cd^	ND	0.097 ± 0.036 ^bc^	ND	0.432 ± 0.11 ^cd^
IG (8 min)-SHS (4 min)-IG (180 °C)	ND	0.331 ± 0.039 ^cd^	ND	0.075 ± 0.003 ^c^	ND	0.406 ± 0.041 ^cd^
IG (8 min)-SHS (4 min)-IG (200 °C)	ND	0.300 ± 0.024 ^d^	ND	0.077 ± 0.03 ^c^	ND	0.377 ± 0.046 ^d^
IG (8 min)-SHS (4 min)-IG (220 °C)	ND	0.418 ± 0.020 ^bc^	ND	0.121 ± 0.021 ^bc^	ND	0.539 ± 0.041 ^bcd^
IG (8 min)-SHS (5 min)-IG (180 °C)	ND	0.269 ± 0.021 ^d^	ND	0.093 ± 0.015 ^bc^	ND	0.362 ± 0.036 ^d^
IG (8 min)-SHS (5 min)-IG (200 °C)	ND	0.364 ± 0.067 ^cd^	ND	0.117 ± 0.054 ^bc^	ND	0.481 ± 0.12 ^cd^
IG (8 min)-SHS (5 min)-IG (220 °C)	ND	0.422 ± 0.028 ^bc^	ND	0.178 ± 0.083 ^ab^	ND	0.600 ± 0.11 ^abc^
IG (8 min)-microwave (1000 w 10 s)-IG (200 °C)	ND	0.294 ± 0.032 ^C^	ND	0.083 ± 0.009 ^B^	ND	0.377 ± 0.039 ^C^
IG (8 min)-microwave (500 w 10 s)-IG (200 °C)	ND	0.462 ± 0.081 ^AB^	ND	0.139 ± 0.013 ^A^	ND	0.601 ± 0.092 ^AB^
IG (8 min)-microwave (500 w 30 s)-IG (200 °C)	ND	0.443 ± 0.051 ^B^	ND	0.119 ± 0.016 ^AB^	ND	0.563 ± 0.063 ^B^
IG (8 min)-microwave (500 w 60 s)-IG (200 °C)	ND	0.325 ± 0.020 ^C^	ND	0.126 ± 0.024 ^A^	ND	0.451 ± 0.042 ^C^
IG (8 min)-microwave (1000 w 10 s)-SHS (4 min)-IG (200 °C)	ND	0.387 ± 0.047 ^z^	ND	0.114 ± 0.027 ^y^	ND	0.501 ± 0.074 ^y^
IG (8 min)-microwave (500 w 10 s)-SHS (4 min)-IG (200 °C)	ND	0.383 ± 0.090 ^z^	ND	0.084 ± 0.003 ^y^	ND	0.467 ± 0.089 ^yz^
IG (8 min)-microwave (500 w 30 s)-SHS (4 min)-IG (200 °C)	ND	0.538 ± 0.004 ^y^	ND	0.120 ± 0.006 ^x^	ND	0.658 ± 0.010 ^x^
IG (8 min)-microwave (500 w 60 s)-SHS (4 min)-IG (200 °C)	ND	0.413 ± 0.048 ^z^	ND	0.114 ± 0.082 ^x^	ND	0.527 ± 0.054 ^y^

Mean ± SD, standard deviation. “CG” means charcoal grilling, “IG” means infrared grilling, and “SHS” means superheated steam roasting. The different letters a–d represent a significant difference between the content of HAs of “IG” and “IG-SHS-IG” grilled patties at *p* < 0.05. The different letters A–C represent a significant difference between the content of HAs of “IG (200 °C)” and “IG-microwave-IG (200 °C)” grilled patties at *p* < 0.05. The different letters x–z represent a significant difference between the content of HAs of “IG (200 °C)” and “IG-microwave-SHS- IG (200 °C)” grilled patties at *p* < 0.05. ND means the content is lower than the detection limit.

**Table 5:** The correlation coefficients between hazards and TBA values, moisture content in patties grilled combined with microwaves or SHS (superheated steam roasting). * means *p* < 0.05, ** means *p* < 0.01.

Techniques	Types of HAs	TBA Value	Moisture Content
Microwave	Norharman	0.408	0.936 **
	Harman	0.541	0.651
SHS	Norharman	0.934 **	0.624
	Harman	0.754 *	0.597

**Table 6:** Effects of different thermal processes on the texture in grilled beef patties.

Cooking Process	Parameter					
	Hardness	Springiness	Cohesiveness	Gumminess	Chewiness	Resilience
CG	6766.43 ± 18.92	0.909 ± 0.02	0.832 ± 0.00	5629.67 ± 21.85	5117.37 ± 18.21	0.417 ± 0.01
IG (180 °C)	4682.62 ± 28.39 ^g^	0.81 ± 0.00 ^e^	0.91 ± 0.02 ^b^	4261.18 ± 21.46 ^d^	3451.56 ± 19.87 ^i^	0.37 ± 0.01 ^bc^
IG (200 °C)	4969.90 ± 28.37 ^dDz^	0.93 ± 0.02 ^dAz^	0.87 ± 0.04 ^cBx^	4323.81 ± 28.37 ^cDz^	4021.14 ± 20.81 ^eEz^	0.40 ± 0.01 ^abAx^
IG (220 °C)	5217.76 ± 34.42 ^b^	1.00 ± 0.01 ^bc^	0.96 ± 0.01 ^a^	5009.05 ± 15.37 ^a^	5009.05 ± 18.01 ^a^	0.40 ± 0.04 ^ab^
IG (8 min)-SHS (3 min)-IG (180 °C)	4781.28 ± 23.91 ^f^	0.93 ± 0.01 ^d^	0.83 ± 0.01 ^d^	3968.46 ± 13.24 ^f^	3690.67 ± 19.82 ^g^	0.35 ± 0.02 ^cd^
IG (8 min)-SHS (3 min)-IG (200 °C)	5001.43 ± 27.18 ^d^	0.99 ± 0.01 ^bc^	0.83 ± 0.00 ^d^	4151.19 ± 18.69 ^e^	4109.68 ± 29.18 ^d^	0.41 ± 0.00 ^a^
IG (8 min)-SHS (3 min)-IG (220 °C)	5481.71 ± 17.29 ^a^	1.03 ± 0.00 ^a^	0.80 ± 0.01 ^e^	4385.37 ± 13.90 ^b^	4516.92 ± 19.04 ^b^	0.43 ± 0.00 ^a^
IG (8 min)-SHS (4 min)-IG (180 °C)	4817.45 ± 27.19 ^f^	0.93 ± 0.02 ^d^	0.78 ± 0.02 ^ef^	3757.61 ± 28.19 ^h^	3494.57 ± 18.23 ^h^	0.34 ± 0.01 ^cd^
IG (8 min)-SHS (4 min)-IG (200 °C)	5061.81 ± 18.02 ^c^	0.98 ± 0.00 ^c^	0.77 ± 0.00 ^f^	3897.59 ± 13.91 ^g^	3819.64 ± 17.26 ^f^	0.40 ± 0.01 ^ab^
IG (8 min)-SHS (4 min)-IG (220 °C)	5497.82 ± 12.93 ^a^	1.01 ± 0.02 ^ab^	0.76 ± 0.01 ^f^	4178.29 ± 28.16 ^e^	4220.13 ± 21.16 ^c^	0.42 ± 0.01 ^a^
IG (8 min)-SHS (5 min)-IG (180 °C)	4908.26 ± 16.26 ^e^	0.95 ± 0.00 ^d^	0.73 ± 0.00 ^g^	3583.03 ± 28.19 ^j^	3403.88 ± 13.02 ^j^	0.33 ± 0.03 ^d^
IG (8 min)-SHS (5 min)-IG (200 °C)	5082.27 ± 18.01 ^c^	1.00 ± 0.03 ^bc^	0.73 ± 0.00 ^g^	3710.06 ± 18.21 ^i^	3710.06 ± 18.27 ^g^	0.42 ± 0.01 ^a^
IG (8 min)-SHS (5 min)-IG (220 °C)	5513.93 ± 17.91 ^a^	1.03 ± 0.01 ^a^	0.71 ± 0.01 ^g^	3914.89 ± 12.13 ^g^	4032.34 ± 16.23 ^e^	0.42 ± 0.00 ^a^
IG (8 min)-microwave (1000 w 10 s)-IG (200 °C)	5312.36 ± 23.19 ^BC^	0.87 ± 0.01 ^B^	0.95 ± 0.01 ^A^	5046.74 ± 13.87 ^B^	4390.67 ± 15.59 ^B^	0.39 ± 0.03 ^A^
IG (8 min)-microwave (500 w 10 s)-IG (200 °C)	5289.27 ± 9.25 ^C^	0.93 ± 0.00 ^A^	0.96 ± 0.00 ^A^	5077.70 ± 14.65 ^AB^	4722.26 ± 15.87 ^A^	0.40 ± 0.03 ^A^
IG (8 min)-microwave (500 w 20 s)-IG (200 °C)	5330.23 ± 10.23 ^B^	0.86 ± 0.00 ^B^	0.94 ± 0.03 ^A^	5010.42 ± 13.86 ^C^	4308.96 ± 24.54 ^C^	0.38 ± 0.01 ^A^
IG (8 min)-microwave (500 w 30 s)-IG (200 °C)	5429.26 ± 18.29 ^A^	0.83 ± 0.01 ^C^	0.94 ± 0.02 ^A^	5103.50 ± 12.87 ^A^	4235.91 ± 14.53 ^D^	0.41 ± 0.03 ^A^
IG (8 min)-microwave (1000 w 10 s)-SHS (4 min)-IG (200 °C)	5419.32 ± 17.28 ^y^	0.97 ± 0.01 ^y^	0.68 ± 0.01 ^z^	4446.74 ± 13.94 ^y^	4390.67 ± 23.98 ^y^	0.34 ± 0.01 ^y^
IG (8 min)-microwave (500 w 10 s)-SHS (4 min)-IG (200 °C)	5137.29 ± 16.39 ^z^	0.95 ± 0.00 ^z^	0.69 ± 0.01 ^z^	4577.70 ± 12.75 ^x^	4722.26 ± 34.72 ^x^	0.31 ± 0.02 ^z^
IG (8 min)-microwave (500 w 20 s)-SHS (4 min)-IG (200 °C)	5440.23 ± 12.38 ^y^	0.96 ± 0.00 ^yz^	0.72 ± 0.02 ^y^	4360.42 ± 17.92 ^z^	4308.96 ± 29.64 ^y^	0.34 ± 0.02 ^y^
IG (8 min)-microwave (500 w 30 s)-SHS (4 min)-IG (200 °C)	5567.29 ± 17.29 ^x^	1.01 ± 0.01 ^x^	0.82 ± 0.01 ^x^	4503.50 ± 18.46 ^x^	4235.91 ± 13.79 ^z^	0.35 ± 0.00 ^y^

Mean ± SD, standard deviation. “CG” means charcoal grilling, “IG” means infrared grilling, and “SHS” means superheated steam roasting. The different letters a–j represent a significant difference between every texture parameter of “IG” and “IG-SHS-IG” grilled patties at *p* < 0.05. The different letters A–E represent a significant difference between every texture parameter of “IG (200 °C)” and “IG-microwave-IG (200 °C)” grilled patties at *p* < 0.05. The different letters x–z represent a significant difference between every texture parameter of “IG (200 °C)” and “IG-microwave-SHS- IG (200 °C)” grilled patties at *p* < 0.05.

## Supplementary Materials

The following are available online at https://www.mdpi.com/article/10.3390/foods10071490/s1, Table S1: The LOQs, LODs and recovery % of UPLC-MS/MS, Table S2: The cooking time of 21 thermal processes.
