# Supplementary material for: Reduction of the Heterocyclic Amines in Grilled Beef Patties through the Combination of Thermal Food Processing Techniques without Destroying the Grilling Quality Characteristics
Source: Foods. 2021 Jun 27;10(7):1490. doi: 10.3390/foods10071490 (PMC8304586; doi:10.3390/foods10071490)
Supplement: Supplementary file 1 [file foods-10-01490-s001.zip › Supplementary (Table S1).pdf]

Supplementary

Table S1. Limit of detection, Limit of quantification and recovery % of UPLC-MS/MS method.

Compound Name	Limit of detection (ng/mL)	Limit of Quantification (ng/mL)	Recovery %
Norharman	0.0038	0.0126	82.23
Harman	0.0041	0.0137	77.90
AαC	0.0023	0.0078	68.57
MeAαC	0.0057	0.0189	95.54
IQx	0.0157	0.0522	69.37
MeIQ	0.0250	0.0834	96.03
MeIQx	0.0172	0.0575	87.2
PhIP	0.0041	0.0137	73.03
7,8-DiMeIQx	0.0058	0.0193	90.27
Trp-P-2	0.0022	0.0072	93.04
4,8-DiMeIQx	0.0076	0.0255	90.96

Recoveries were performed by adding 50, 100, and 150 μL of 200 ng/mL mixed standard solutions to control samples. The results of recoveries given in % values of all HAAs.
