# Supplementary material for: Reduction of the Heterocyclic Amines in Grilled Beef Patties through the Combination of Thermal Food Processing Techniques without Destroying the Grilling Quality Characteristics
Source: Foods. 2021 Jun 27;10(7):1490. doi: 10.3390/foods10071490 (PMC8304586; doi:10.3390/foods10071490)
Supplement: Supplementary file 1 [file foods-10-01490-s001.zip › Supplementary (Table S2).pdf]

Supplementary

Table S2. The cooking time of grilled beef patties heated by 21 thermal processes.

Thermal processes	Cooking time (s)
CG	$872±21.29^{h}$
IG (180℃)	$1554±98.86^{a}$
IG (200℃)	$1651±88.13^{a}$
IG (220℃)	$1504±62.07^{b}$
IG (8min) - SHS (3 min) - IG (180℃)	$1437±47.67^{b}$
IG (8min) - SHS (3min) - IG (200℃)	$1423±51.26^{c}$
IG (8min) - SHS (3min) - IG (220℃)	$1411±199.8^{c}$
IG (8min) - SHS (4min) - IG (180℃)	$1322±315.93^{d}$
IG (8min) - SHS (4min) - IG (200℃)	$1399±66.82^{c}$
IG (8min) - SHS (4min) - IG (220℃)	$1478±33.35^{b}$
IG (8min) - SHS (5min) - IG (180℃)	$1281±176.13^{d}$
IG (8min) - SHS (5min) - IG (200℃)	$1314±116.46^{d}$
IG (8min) - SHS (5min) - IG (220℃)	$1364±33.74^{dc}$
IG (8min) - microwave (1000w 10s) - IG (200℃)	$1316±68.08^{c}$
IG (8min) - microwave (500w 10s) - IG (200℃)	$1511±42.67^{b}$
IG (8min) - microwave (500w 20s) - IG (200℃)	$1401±206.32^{c}$
IG (8min) - microwave (500w 30s) - IG (200℃)	$1216±90.12^{e}$
IG (8min) - microwave (1000w 10s) - SHS (4min) - IG (200℃)	$1312±53.1^{d}$
IG (8min) - microwave (500w 10s) - SHS (4min) - IG (200℃)	$1435±10.5^{c}$
IG (8min) - microwave (500w 20s) - SHS (4min) - IG (200℃)	$1336±36.25^{e}$
IG (8min) - microwave (500w 30s) - SHS (4min) - IG (200℃)	$1016±116.4^{g}$

Mean ± SD. Different letters represent significant differences under different thermal processes ($P<0.05$).
